# Inhibition of Development and Metabolism of Dual-Species Biofilms of *Candida albicans* and *Candida krusei* (*Pichia kudriavzevii*) by Organoselenium Compounds

**DOI:** 10.3390/ph17081078

**Published:** 2024-08-16

**Authors:** Gabriela de Souza Calvi, Giulia Nicolle Jácome Cartaxo, Qiuxin Lin Carretoni, André Luiz Missio da Silva, Denilson Nogueira de Moraes, José Geraldo da Cruz Pradella, Maricilia Silva Costa

**Affiliations:** Instituto de Pesquisa & Desenvolvimento—IP&D, Universidade do Vale do Paraíba—UNIVAP, Av. Shishima Hifumi, 2911, São José dos Campos 12244-390, SP, Brazil; gabriela.silva753.gs@gmail.com (G.d.S.C.); giuliacartaxo@gmail.com (G.N.J.C.); joycarretoni@gmail.com (Q.L.C.); andremissio2003@gmail.com (A.L.M.d.S.); dnmoraes@univap.br (D.N.d.M.); jpradella51@gmail.com (J.G.d.C.P.)

**Keywords:** *Candida albicans*, *Candida krusei*, *Pichia kudriavzevii*, biofilms, organoselenium compounds, antifungal therapy, antifungal resistance

## Abstract

Although *Candida albicans* is the most frequently identified *Candida* species in clinical settings, a significant number of infections related to the non-*albicans Candida* (NAC) species, *Candida krusei*, has been reported. Both species are able to produce biofilms and have been an important resistance-related factor to antimicrobial resistance. In addition, the microbial relationship is common in the human body, contributing to the formation of polymicrobial biofilms. Considering the great number of reports showing the increase in cases of resistance to the available antifungal drugs, the development of new and effective antifungal agents is critical. The inhibitory effect of Organoselenium Compounds (OCs) on the development of *Candida albicans* and *Candida krusei* was recently demonstrated, supporting the potential of these compounds as efficient antifungal drugs. In addition, OCs were able to reduce the viability and the development of biofilms, a very important step in colonization and infection caused by fungi. Thus, the objective of this study was to investigate the effect of the Organoselenium Compounds (*p*-MeOPhSe)_2_, (PhSe)_2,_ and (*p*-Cl-PhSe)_2_ on the development of dual-species biofilms of *Candida albicans* and *Candida krusei* produced using either RPMI-1640 or Sabouraud Dextrose Broth (SDB) media. The development of dual-species biofilms was evaluated by the determination of both metabolic activity, using a metabolic assay based on the reduction of XTT (2,3-bis(2-methoxy-4-nitro-5-sulfophenyl)-2H-tetrazolium-5-carboxanilide sodium salt) assay and identification of either *Candida albicans* and *Candida krusei* on CHROMagar Candida medium. Biofilm formation using RPMI-1640 was inhibited in 90, 55, and 20% by 30 µM (*p*-MeOPhSe)_2_, (PhSe)_2,_ and (*p*-Cl-PhSe)_2_, respectively. However, biofilms produced using SDB presented an inhibition of 62, 30 and 15% in the presence of 30 µM (*p*-MeOPhSe)_2_, (PhSe)_2,_ and (*p*-Cl-PhSe)_2_, respectively. The metabolic activity of 24 h biofilms was inhibited by 35, 30 and 20% by 30 µM (*p*-MeOPhSe)_2_, (PhSe)_2,_ and (*p*-Cl-PhSe)_2_, respectively, with RPMI-1640; however, 24 h biofilms formed using SDB were not modified by the OCs. In addition, a great reduction in the number of CFUs of *Candida albicans* (93%) in biofilms produced using RPMI-1640 in the presence of 30 µM (*p*-MeOPhSe)_2_ was observed. However, biofilms formed using SDB and treated with 30 µM (*p*-MeOPhSe)_2_ presented a reduction of 97 and 69% in the number of CFUs of *Candida albicans* and *Candida krusei*, respectively. These results demonstrated that Organoselenium Compounds, mainly (*p*-MeOPhSe)_2,_ are able to decrease the metabolic activity of dual-species biofilms by reducing both *Candida albicans* and *Candida krusei* cell number during biofilm formation using either RPMI-1640 or SDB. Taken together, these results demonstrated the potential of the OCs to inhibit the development of dual-species biofilms of *Candida albicans* and *Candida krusei*.

## 1. Introduction

Fungi from the *Candida* genus are generally commensal and constitute the regular human microbiota; however, they have the ability to become opportunistic pathogens, mainly in immunocompromised patients, representing a public health challenge [1,2]. *Candida albicans* is frequently involved in fungi-related systemic infections due to its ability to transition from planktonic cell form to filamentous form [3]. The presence of filamentous structures represents an advantage for the fungus, contributing to its adhesion to the substrate and, consequently, to the formation of a complex filamentous system called biofilm [4,5]. Although *Candida albicans* is the most frequently identified *Candida* species in clinical contexts, a significant number of infections related to the non-*albicans Candida* (NAC) species, such as *Candida krusei* (*Pichia kudriavzevii*), has been reported [6,7]. In addition, mortality levels of infections caused by *Candida krusei* are alarming, mainly due to its natural resistance to fluconazole—a first-choice antifungal for the treatment of candidiasis [8,9].

Similar to *Candida albicans*, *Candida krusei* is also able to produce biofilms, increasing the resistance to conventional antifungal therapy and, consequently, extending the infections [10,11,12]. Biofilm formation is a complex process presenting distinct phases of development: adhesion to an abiotic or biotic surface, cell proliferation and early-stage filamentation of the adhered cells, and, finally, biofilm maturation [4]. Biofilms are communities of polymorphic cells, including hyphal cells, pseudohyphal cells, and yeast cells, protected by an extracellular matrix (ECM) that not only allows fungi to survive in critical conditions but also permits the cells in the biofilm structure to escape the immune system’s defenses [11,13]. In the medical context, biofilms can colonize diverse abiotic surfaces, such as catheters and dentures, although biotic surfaces, such as the gastrointestinal tract, female reproductive tract, oral cavity, and skin, can provide a propitious surface to biofilm development [4,14,15,16].

One of the main causes of nosocomial infections worldwide is those related to Candida species, mainly in immunocompromised patients [17]. In addition, it has been demonstrated that Candidaemia is the principal form of invasive candidiasis, representing the fourth cause of bloodstream infections in intensive care unit settings with high mortality rates [18,19].

Furthermore, an increase in the number of studies demonstrating the increase in rates of antifungal resistance in most fungal infections has been observed [20,21]. The first Fungal Priority Pathogens List (WHO FPPL) was published in 2022 to direct research, development, and public health actions to reinforce the response to antifungal resistance. This list classifies the fungal pathogens into three priority categories: critical, high, and medium. It is very important to highlight that the species *Candida albicans* and *Candida krusei* (*Pichia kudriavzevii*) were included in the WHO FPPL, with *Candida albicans* categorized into critical groups. In this context, there is an urgent need to develop effective strategies and procedures to control opportunistic human pathogens.

Organoselenium Compounds (OCs) are important intermediates and reagents used in organic syntheses [22] with applications in diverse biological processes, presenting neuroprotective, antitumor, and antimicrobial actions, also acting as cytokine inducers and immunomodulators of the central nervous system [23,24]. The antimicrobial activity of OCs against pathogenic fungi and bacteria has been described by different authors [25,26,27,28]. In the last years, our group has been studying the potential of OCs (*p*-MeOPhSe)_2_, (PhSe)_2,_ and (*p*-Cl-PhSe)_2_ as promising antifungal drugs against both *Candida albicans* and *Candida* [29,30,31,32,33]. These studies demonstrated the ability of OCs (*p*-MeOPhSe)_2_, (PhSe)_2,_ and (*p*-Cl-PhSe)_2_ to inhibit the development of both *Candida albicans* and *Candida krusei*, decreasing biofilm formation and also the viability of formed biofilms.

The human microbiota is composed of a collection of dynamic microbial communities, and it is common to find diverse microorganisms coexisting [34]. Interactions between different microorganisms are complex and can be synergistic, antagonistic, or present neutral relationships [13]. Furthermore, multispecies communities can produce polymicrobial biofilms that can lead to persistent infections in different sites in the human body [34,35]. These polymicrobial infections represent an additional challenge due to the need to administer higher doses of antimicrobial medications, increasing the risks for the patient since it is known that some antifungal drugs can damage kidney and liver cells, mainly when used for extended periods [36]. Consequently, studies related to the development of new and efficient antifungal treatments against multispecies biofilms are crucial. Thus, the objective of this study was to determine the effect of OCs (*p*-MeOPhSe)_2_, (PhSe)_2,_ and (*p*-Cl-PhSe)_2_ on the development of dual-species biofilms of *Candida albicans* and *Candida krusei*.

## 2. Results

**Figure 1:**
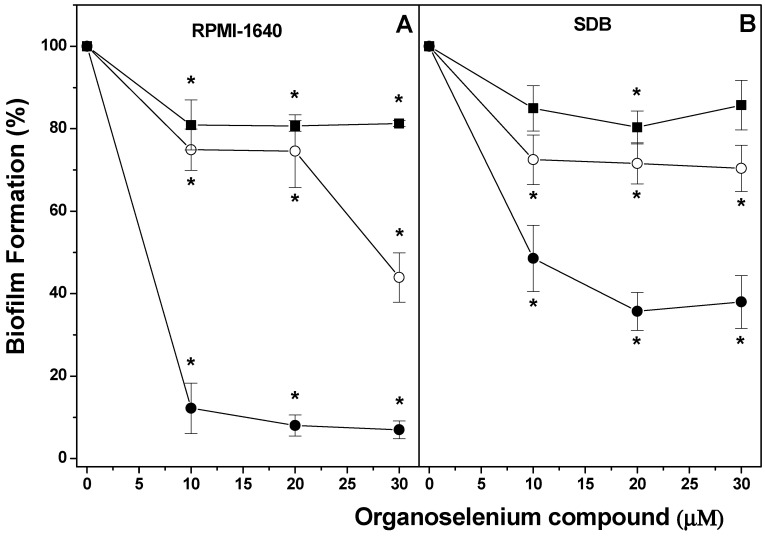
Effect of (*p*-MeOPhSe)_2_ (●), (PhSe)_2_ (ο), and (*p*-Cl-PhSe)_2_ (^▄^) on the metabolic activity of dual-species biofilm in formation using either RPMI-1640 (**A**) or SDB (**B**) medium. Details of the experimental conditions are described in Methods. Yeast-mixed suspension was used to form biofilms for 24 h in the presence of different OC concentrations, with RPMI-1640 or SDB. After this period, the metabolic activity of dual-species biofilms was determined using XTT. The values presented in the figure represent the percentage of the metabolic activity of biofilms, calculated using the control group (biofilms formed in the absence of OCs) as 100% of the metabolic activity of biofilms. The data are mean ± SE (*n* = 8). * *p* < 0.05.

The effect of different Organoselenium Compounds (OCs) was determined on the formation of dual-species biofilm of *Candida albicans* and *Candida krusei*, using either RPMI-1640 or SDB (Figure 1). Biofilm formation using RPMI-1640 was inhibited in the presence of (*p*-MeOPhSe)_2_ in concentrations varying from 10 to 30 µM (~90%). The compounds (PhSe)_2_ and (*p*-Cl-PhSe)_2_ were also able to reduce biofilm formation but to a minor extent. Inhibition of ~55% and ~20% in the presence of 30 µM (PhSe)_2_ and (*p*-Cl-PhSe)_2_ was observed, respectively (Figure 1A). However, biofilms produced using SDB presented a minor inhibition by OCs. Inhibition of 62, 30, and 15% in the presence of 30 µM (*p*-MeOPhSe)_2_, (PhSe)_2,_ and (*p*-Cl-PhSe)_2_ was observed, respectively (Figure 1B).

**Figure 2:**
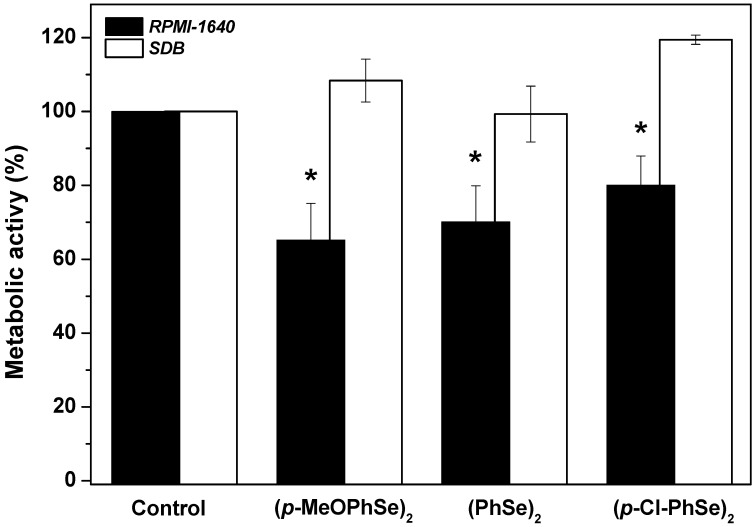
Effect of (*p*-MeOPhSe)_2_, (PhSe)_2,_ and (*p*-Cl-PhSe)_2_ on the metabolic activity of 24 h dual-species biofilm. Details of the experimental conditions are described in Methods. Yeast-mixed suspension was used to produce biofilms for 24 h in the presence of RPMI-1640 or SDB. After this period, biofilms were treated and grown for an additional 24 h in the presence of 30 μM OCs with either RPMI-1640 or SDB. The values presented in the figure represent the percentage of the metabolic activity of biofilms, calculated using the control group (biofilms formed in the absence of OCs) as 100% of the metabolic activity of biofilms. The data are mean ± SE (*n* = 8). * *p* < 0.05.

Figure 2 shows the effect of 30 µM (*p*-MeOPhSe)_2_, (PhSe)_2,_ and (*p*-Cl-PhSe)_2_ on 24 h dual-species biofilm produced using either RPMI-1640 or SDB. When studying the effect of the compounds on biofilms produced over 24 h, it was observed that concentrations lower than 30 µM were not able to produce important inhibitory effects on metabolic activity. For this reason, it was chosen the concentration of 30 µM since it is not an exceedingly high concentration, a fact that could not be positive when choosing a medicine. An inhibition of 35, 30, and 20% in the metabolic activity of 24 h dual-species biofilm produced using RPMI-1640 and treated for additional 24 h with 30 µM (*p*-MeOPhSe)_2_, (PhSe)_2,_ and (*p*-Cl-PhSe)_2_, respectively, was observed. However, the 24 h dual-species biofilm of *Candida albicans* and *Candida krusei* formed using SDB was not modified by the OCs tested. Taken together, these results demonstrated the potential of the OCs to inhibit the development of dual-species biofilms of *Candida albicans* and *Candida krusei* and that the compounds are more effective on biofilms produced using RPMI-1640.

**Figure 3:**
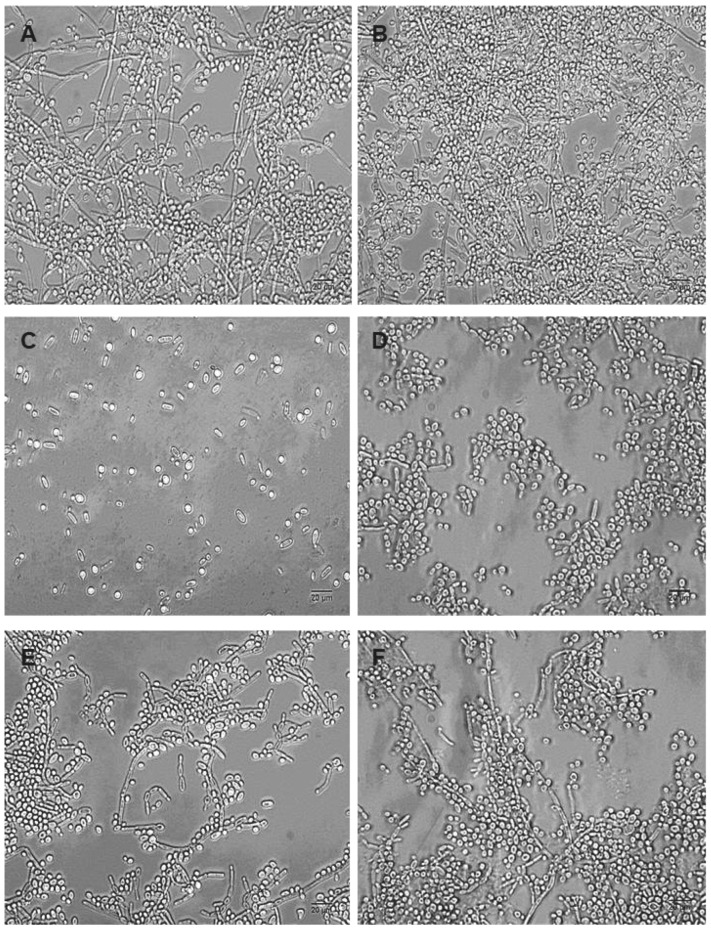
Morphological characterization of dual-species biofilms of *Candida albicans* and *Candida krusei*. Yeast-mixed suspensions were used to form biofilms in the absence (**A**,**B**) and in the presence of 30 µM (*p*-MeOPhSe)_2_ (**C**,**D**) or 30 µM (PhSe)_2_ (**E**,**F**). (**A**,**C**,**E**) represents biofilms formed during 24 h in the presence of OCs. (**B**,**D**,**F**) represents 24 h biofilms treated with OCs. Bar 20 µm.

The inhibitory effect of OCs can also be observed when observing the morphology of the biofilms produced using RPMI-1640 by light microscopy (Figure 3).

Dual-species biofilms produced using RPMI-1640 presented a large amount of cells in the filamentous form, and cells were observed in both spherical and cylindrical forms, typical of *Candida albicans* and *Candida krusei*, respectively (Figure 3A). Compared to the control group, biofilms produced in the presence of 30 µM (*p*-MeOPhSe)_2_ and (PhSe)_2_ presented an evident decrease in the number of cells in both yeast and filamentous form (compare Figure 3C,E with Figure 3A). However, the total absence of cells presenting filamentous form was observed in dual-species biofilms produced in the presence of 30 µM (*p*-MeOPhSe)_2_. Despite having a smaller effect than (*p*-MeOPhSe)_2_, the compound (PhSe)_2_ was able to reduce the number of cells in either yeast or filamentous form in biofilm formation. In addition, a reduction in the length of the filaments was observed in biofilms formed in the presence of 30 µM (PhSe)_2_ (Figure 3E). However, 24 h dual-species biofilms were less affected by OCs than biofilms in formation. Biofilms produced and posteriorly treated with 30 µM (*p*-MeOPhSe)_2_ or (PhSe)_2_ for an additional 24 h presented a reduction in the number of cells (compare Figure 3D,F with Figure 3B). The number of cells presenting filamentous forms was greatly reduced after treatment with 30 µM (*p*-MeOPhSe)_2_ (Figure 3D). These results support the data presented in Figure 1 and Figure 2, showing the reduction in both metabolic activity and cell number in dual-species biofilms.

**Figure 4:**
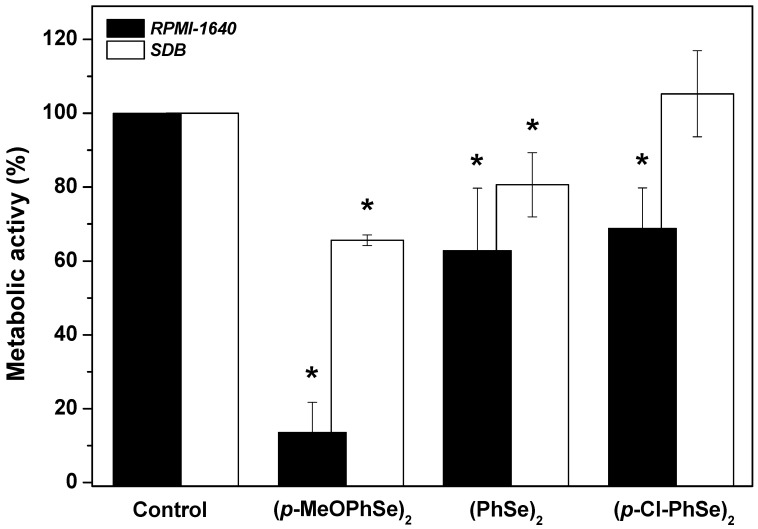
Effect of (*p*-MeOPhSe)_2_, (PhSe)_2,_ and (*p*-Cl-PhSe)_2_ on cell adhesion to polystyrene surfaces. Details of the experimental conditions are described in Methods. Values presented in the figure represent the percentage of the metabolic activity of biofilms, calculated using the control group (biofilms formed in the absence of OCs) as 100% of the metabolic activity of biofilms. The data are mean ± SE (*n* = 8). * *p* < 0.05.

In order to understand in which of the biofilm formation stages the OCs act, the effect of OCs was determined on cell adhesion (Figure 4). Following the period of cell adhesion, biofilm formation was produced using either RPMI-1640 or SDB. A significant reduction in biofilm formation using RPMI-1640 when cell adhesion was formed in the presence of 30 µM (*p*-MeOPhSe)_2_ was observed. The compounds (PhSe)_2_ e (*p*-Cl-PhSe)_2_ were also able to reduce the cell adhesion but to a minor extent. Biofilms formed using RPMI-1640 after a period of adhesion in the presence of 30 µM (*p*-MeOPhSe)_2_, (PhSe)_2,_ and (*p*-Cl-PhSe)_2_ presented a decrease of 86, 37 and 31% in metabolic activity, respectively. However, after the period of adhesion in the presence of 30 µM (*p*-MeOPhSe)_2_ or (PhSe)_2_, biofilms formed using SDB presented a decrease of 35 and 20% in the metabolic activity, respectively. The presence of (*p*-Cl-PhSe)_2_ during the adhesion period did not modify the metabolic activity of biofilms formed using SDB.

**Figure 5:**
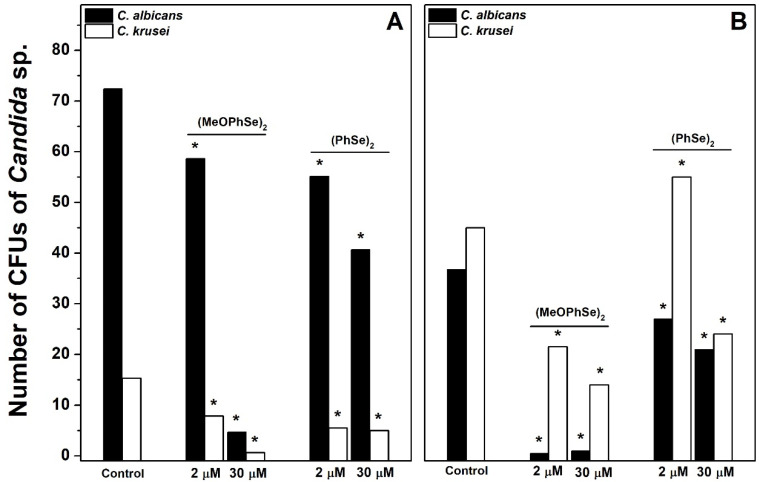
Effect of (*p*-MeOPhSe)_2_ and (PhSe)_2_ on the number of CFUs of *Candida albicans* and *Candida krusei* in biofilm formation. The experimental conditions are described under Materials and Methods. Yeast-mixed suspension was used to produce biofilms for 24 h in the presence of RPMI-1640 (**A**) or SDB (**B**). The number of CFUs of both *Candida albicans* and *Candida krusei* was determined after different treatments using CHROMagar Candida medium. The data are mean ± SE (*n* = 8). * *p* < 0.05.

Following, to determine the contribution of each species in metabolic activity obtained, the number of CFUs of both *Candida albicans* and Candida *krusei* was determined after different treatments. A high *Candida albicans*/*Candida krusei* ratio in biofilms formed using RPMI-1640 was observed, while a repeated predominance of *Candida krusei* was observed in biofilms formed using SDB (Figure 5).

**Figure 6:**
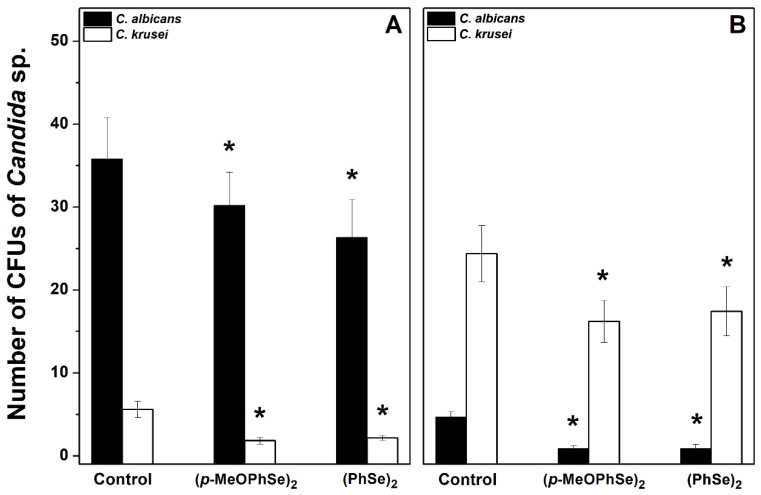
Effect of (*p*-MeOPhSe)_2_ and (PhSe)_2_ on the number of CFUs of *Candida albicans* and *Candida krusei* in 24 h dual-species biofilms cultivated on either RPMI-1640 (**A**) or SDB (**B**). Experimental conditions are described in Methods. The number of CFUs of both *Candida albicans* and *Candida krusei* was determined after different treatments using CHROMagar Candida medium. The data are mean ± SE (*n* = 8). * *p* < 0.05.

This result demonstrates that different *Candida albicans*/*Candida krusei* ratios are present in biofilms formed using either RPMI-1640 or SDB. In biofilms formed using RPMI-1640, the number of CFUs of *Candida albicans* was 72, 59, and 5 in the absence and in the presence of 2 and 30 µM (*p*-MeOPhSe)_2_, respectively (Figure 5A). Furthermore, the number of CFUs of *Candida albicans* was 37, 0.5, and 1 in the absence and in the presence of 2 and 30 µM (*p*-MeOPhSe)_2_, respectively, in biofilms formed using SDB (Figure 5B). A decrease in the number of CFUs of *Candida albicans* by 30 µM (PhSe)_2_ was observed, but this effect was smaller than that obtained with 30 µM (*p*-MeOPhSe)_2_, using either RPMI-1640 (43%) or SDB (42%). (*p*-MeOPhSe)_2_ was also able to reduce the number of CFUs of *Candida krusei*; however, the impact of this inhibition in total metabolic activity was lower, probably due to the limited *Candida krusei* collaboration in the structure of biofilm produced using RPMI-1640. Furthermore, biofilms formed using SDB and treated with 30 µM (*p*-MeOPhSe)_2_ presented a reduction of 97 and 69% in the number of CFUs of *Candida albicans* and *Candida krusei*, respectively. These results demonstrated that (*p*-MeOPhSe)_2_ was able to decrease the metabolic activity of dual-species biofilms by reducing the cell number of both *Candida albicans* and *Candida krusei* during biofilm formation using either RPMI-1640 or SDB. Figure 6 shows the number of CFUs of *Candida albicans* and *Candida krusei* in 24 h dual-species biofilms produced using either RPMI-1640 or SDB and treated for an additional 24 h with 30 µM (*p*-MeOPhSe)_2_ or (PhSe)_2_. Similar to what is shown in Figure 5, different *Candida albicans*/*Candida krusei* ratios were obtained using either RPMI-1640 or SDB. A Candida albicans/Candida krusei ratio of 6.3 and 0.2 was observed in biofilms produced using RPMI-1640 or SDB, respectively. In 24 h dual-species biofilms produced using RPMI-1640 it was observed a reduction in the number of CFUs of *Candida albicans* of 16 and 27% in the presence of 30 µM (*p*-MeOPhSe)_2_ and (PhSe)_2_, respectively; however, the number of CFUs of *Candida krusei* was reduced around 64% in the presence of either 30 µM (*p*-MeOPhSe)_2_ or (PhSe)_2_ (Figure 6A).

Biofilms formed using SDB presented a decrease in the number of CFUs of *Candida albicans* and *Candida krusei* of 78 and 31%, respectively, by either 30 µM (*p*-MeOPhSe)_2_ or (PhSe)_2_ (Figure 6B). Taken together, these results demonstrated the potential of the Organoselenium Compounds as promisor new antifungal drugs.

## 3. Discussions

Despite its lower frequency, invasive and/or systemic candidiasis present higher mortality (35 to 80%), producing around 1.5 million deaths per year in the world [37]. Furthermore, an increase in the incidence of Candidemias has been described by different authors [38,39]. This problem became more evident after the COVID-19 pandemic because of the increase in the cases of COVID-19-associated candidiasis worldwide [37,40]. In this context, and considering the great number of reports showing the increase in the cases of resistance to the available antifungal drugs, the development of new and effective antifungal agents is critical. The potential of different Organoselenium Compounds as promising antifungal drugs has been demonstrated. The ability of different OCs to reduce the development of biofilms mono-species of either *Candida albicans* or *Candia krusei* was recently demonstrated [30,31,32,33]. In this paper, we demonstrated, for the first time, the inhibitory effect of (*p*-MeOPhSe)_2_, (PhSe)_2,_ and (*p*-Cl-PhSe)_2_ on dual-species biofilm of *Candida albicans* and *Candida krusei*. Although, the inhibitory effect was clearly dependent on the medium used to form biofilms. The results presented here showed that dual-species biofilms produced using RPMI-1640 present a predominance of *Candida albicans* on *Candida krusei* cells and, consequently, a high *Candida albicans*/*Candida krusei* ratio, while biofilms formed with SDB showed the greater number of *Candida krusei* than *Candida albicans* cells. This alteration in biofilm structure was also observed when analyzing the morphology of cells in biofilms by light microscopy. Thus, the differences observed in the inhibitory effect seem to be related to different *Candida albicans*/*Candida krusei* ratios obtained when using different media. It was recently demonstrated that the inhibitory effect of antimicrobial photodynamic therapy (aPDT) on dual-species biofilms of *Candida albicans* and *Candida krusei* can be modified by *Candida albicans*/*Candida krusei* ratio and that *Candida krusei*, when present in the structure of dual-species biofilms can be resistant to aPDT [41]. Furthermore, it was demonstrated that the medium and environment could regulate interactions between both yeast species, including the response to Voriconazole [42]. These results indicate that the inhibitory effects of antifungal drugs may be different depending on the medium tested, representing a new challenge in the study of antifungal compounds. A similar effect was demonstrated in this paper since dual-species biofilms formed using RPMI-1640, presenting a high *Candida albicans*/*Candida krusei* ratio, seem to be more sensitive to OCs than biofilms produced using SDB, suggesting a higher resistance of *Candida krusei* when associated with *Candida albicans* in the biofilm structure. However, the inhibitory effect of (*p*-MeOPhSe)_2_ on mono-species biofilms of *Candida albicans* is similar to that observed on *Candida krusei* biofilm formation (~80%) [30,32], indicating that *Candida krusei* when associated with *Candida albicans* in biofilms present tolerance to (*p*-MeOPhSe)_2_. Increased OC concentrations were able to reduce both biofilm formation and viability of 24 h dual-species biofilms, being (*p*-MeOPhSe)_2_ more efficient than (PhSe)_2_ and (*p*-Cl-PhSe)_2_, a similar result to that obtained when studying biofilms mono-species of *Candida albicans* and *Candida krusei*.

The exact mechanism by which OCs decrease the development of both mono and dual-species biofilms of *Candida albicans* and *Candida krusei* is still unknown, although the results obtained in our study suggest that the substituent groups in symmetric diselenides significantly influence their chemical activity. Our experiments demonstrate that deactivating groups, such as chloride, reduce chemical activity, whereas activating groups, such as methoxy, enhance it. Multiple observations indicate that the substituent groups on the aromatic ring play a crucial role in modulating the chemical reactivity of the organoselenium compound [43]. This modulation is mainly attributed to variations in electronegativity relative to carbon, along with differences in atomic volume and spatial arrangements [44].

Furthermore, dual-species biofilms previously formed were more resistant to OCs than biofilms in formation, similar to that observed in mono-species of either *Candida albicans* or *Candida krusei* [30,32]. Žiemytė and collaborators (2023) demonstrated that micafungin and caspofungin failed to eradicate 24 h mono-species biofilms of *Candida albicans* and *Candida glabrata*, corroborating that *Candida* spp. biofilms previously formed are extremely difficult to eliminate [45]. In addition, our results demonstrated that biofilms presenting low *Candida albicans*/*Candida krusei* ratio were refractory to OC treatment. Comparing Figure 1 and Figure 4, it is possible to observe a similarity, suggesting that the inhibitory effect of OCs could be related to a decrease in cell adherence. A significant decrease in *Candida spp*. adhesion to both polystyrene plates and epithelial cells by (PhSe)_2_ and (*p*-Cl-PhSe)_2_ was recently demonstrated [31,33]. Considering that adherence of yeast cells to the epithelial cell surface is the first step for colonization, tissue invasion, and spread of systemic infection [4], drugs able to reduce/eliminate yeast–surface interaction can represent a very important step in the fight against systemic candidiasis. Furthermore, the determination of the number of CFUs of *Candida albicans* and *Candida krusei* in biofilms dual-species demonstrated that the composition of biofilms is a determinant factor in antifungal therapy. In biofilms formed with RPMI-1640, (*p*-MeOPhSe)_2_ decreased the metabolic activity of biofilm formation (~90%) by reducing both *Candida albicans* and *Candida krusei* development, however considering that *Candida albicans* was present in a higher number, the participation of *Candida krusei* in metabolic activity is probably lower. In addition, during biofilm formation with SDB, the participation of *Candida krusei* is higher, and consequently, the inhibition of metabolic activity by (*p*-MeOPhSe)_2_ is lower. Analyzing the number of CFUs of *Candida albicans* and *Candida krusei* in 24 h dual-species biofilms, a similar profile was observed, although inhibition by OCs was significantly reduced. When we reflect on the fight against diseases caused by fungi in the world, some points are most relevant: (1) the increase in the frequency of fungal infections; (2) the increase in the number of infections refractory to medicines available; (3) the toxic effects associated with antifungal drugs. Although amphotericin B (AmB) is a life-saving medicine in the treatment of serious systemic fungal infections and is the most used antifungal medicine in intensive care, the acute and chronic toxicity induced by this medicine represents a limitation in its use [46]. Reactions such as fever, rigors, headache, arthralgia, nausea, vomiting, and hypotension have been related to the AmB administration. Furthermore, the nephrotoxic effect of AmB has been extensively demonstrated [47]. These factors highlighted above make clear the urgency of new treatments against pathogenic fungi. The results present here demonstrate the potential of OCs, mainly (*p*-MeOPhSe)_2,_ as promising antifungal drugs by reducing biofilm formation, viability of biofilm produced, and adhesion to a surface.

## 4. Materials and Methods

### 4.1. Yeast Strain and Growth Conditions

*Candida albicans* (Manassas, VA, USA; ATCC #1023) and *Candida krusei* (Manassas, VA, USA; ATCC #6258) were purchased in American Type Culture Collection and plated on Sabouraud dextrose agar (Merck, Darmstadt, Hesse, Germany). Cultures were incubated in ambient air for 48 h at 37 °C. After this period, a sample of the cultures was removed from the surface of the agar and suspended in a physiological solution at a cell density of 10^7^ cell.mL^−1^, determined using the Neubauer chamber.

### 4.2. Organoselenium Compounds (OCs)

**Figure 7:**
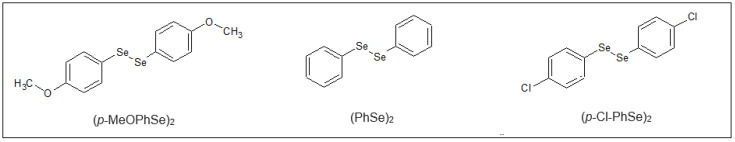
Chemical structures of the Organoselenium Compounds. *p*-chloro-diphenyl diselenide-(*p*-Cl-PhSe)_2;_ *p*-methoxy-diphenyl diselenide-(*p*-MeOPhSe)_2_; diphenyl diselenide-(PhSe)_2_.

Diphenyl diselenide-(PhSe)_2_ was purchased from Sigma-Aldrich (St. Louis, MO, USA). The compounds *p*-chloro diphenyl diselenide-(*p*-Cl-PhSe)_2_ and p,p′-methoxyl-diphenyl diselenide-(*p*-MeOPhSe)_2_ were obtained according to Paulmier (1986) [48] and were kindly provided by Prof. João Batista Teixeira da Rocha (Universidade Federal de Santa Maria, Brazil). Analysis of the ^1^H NMR and ^13^C NMR spectra showed that compounds presented analytical and spectroscopic data in full agreement with their assigned structure. The chemical purity of all compounds was higher than 99.9%, as determined by GC/HPLC. The chemical structures of the OCs are shown in Figure 7. OC stock solutions were prepared in 100% Dimethylsulphoxide (DMSO; Sigma-Aldrich, St. Louis, MO, USA) and diluted in ultrapure water to reach the test concentration of 1%. The control group was included in all experiments and represented the treatment condition with DMSO 1%, which did not modify the studied parameter in *Candida albicans* and *Candida krusei*.

### 4.3. Effect of OCs on Biofilm Formation

A mixture of suspensions of *Candida albicans* (10^7^ cell·mL^−1^) and *Candida krusei* (10^7^ cell·mL^−1^) was performed in equal proportion (1:1) and used to produce biofilms. This yeast-mixed suspension (25 µL) was seeded in a 96-well polystyrene plate containing either RPMI-1640 medium (Sigma, St. Louis, MO, USA) or Sabouraud Dextrose Broth (SDB) medium (Merck, Darmstadt, Hesse, Germany) in the presence of different concentrations of OCs (varying from 10 to 30 μΜ) in a final volume of 200 μL, for 24 h at 37 °C. After this period, the medium was removed, and each well was washed twice with PBS. Biofilms produced using RPMI-1640 medium were washed with 200 µL, although biofilms produced using SDB medium were washed with 100 µL. Biofilm formation was monitored by XTT assay based on the reduction of XTT (2,3-bis(2-methoxy-4-nitro-5-sulfophenyl)-2H-tetrazolium-5-carboxanilide sodium salt; Molecular Probes, Eugene, OR, USA) assay, according to the literature (da Silva et al., 2021). The reduced formazan-colored product was determined at 490 nm (OD_490_) in a Synergy HT Multi-Detection Microplate Reader (Bio-Tek, Winooski, VT, USA). Data obtained were expressed as a percentage (%) of the metabolic activity of biofilms produced relative to the control group (100%).

### 4.4. Effect of OCs on Dual-Species Biofilm Produced during 24 h

Yeast-mixed suspension (25 µL) was seeded in a 96-well plate containing either RPMI-1640 or SDB in a final volume of 200 μL in the absence of OCs. Plates were incubated for 24 h at 37 °C to produce biofilms. After biofilm formation, the media were removed, the wells were washed three times with PBS solution, and a fresh medium containing 30 µM OCs was added to the wells (final volume = 200 μL). Biofilms were produced for an additional 24 h at 37 °C. Next, the wells were washed twice with PBS, and the metabolic activity of the biofilm produced was monitored by XTT assay.

### 4.5. Effect of OCs on Cell Adhesion to Polystyrene Surfaces

Aliquots of yeast-mixed suspension (25 µL) were added in 96-well plates with saline solution and 30 µM OCs for 90 min at 37 °C. After this period of cell adhesion, the medium was removed, and the wells were washed with PBS (100 µL). Following, either RPMI-1640 or SDB was added to the wells in a final volume of 200 μL, and the plates were incubated for 24 h at 37 °C. Next, the biofilms produced were washed twice with PBS, and the metabolic activity of the biofilm produced was monitored by XTT assay.

### 4.6. Effect of OCs on Number of Colony Forming Units (CFUs) of Either Candida albicans or Candida krusei

In parallel with carrying out the experiments to determine metabolic activity using XTT assay, the number of CFUs of *Candida albicans* and *Candida krusei* present in biofilms structure was determined using CHROMagar Candida^®^ (BD, Sparks, NV, USA). The cells of the biofilms produced after the different treatments using OCs were washed twice with PBS, harvested using a scraper, and diluted 800 times. An aliquot of this biofilm suspension (25 µL) was seeded on CHROMagar Candida medium to determine the number of CFUs of both *Candida albicans* and *Candida krusei* after 48 h at 37 °C. The number of colonies was determined visually.

### 4.7. Morphological Analyses of Dual-Species Biofilms

The morphology of dual-species biofilms was observed by light microscopy before cells in biofilms were harvested. Biofilms were examined using a microscope by light microscopy (Axioskop 2, Zeiss, Jena, Germany), and the images were captured with a Pixera digital camera system (Pixera Corporation, Santa Clara, CA, USA) attached to the photomicroscope and a microcomputer (IntelVR PentiumVR, Santa Clara, CA, USA) using the software Adobe Photoshop version 7.0.1 (Adobe Systems, Atlanta, GA, USA).

### 4.8. Statistical Analysis

Values were expressed as means ± standard deviation (SD) of different and independent experiments (*n* = 8), performed in triplicate. Statistical analyses used was the one-way analysis of variance (ANOVA) followed by the Tukey–Kramer post hoc test for multiple comparisons. *p* values < 0.05 were considered significant. Graphics were presented, and statistical analysis was performed using OriginPro 8.5 (OriginLab Corporation, Northampton, MA, USA).

## 5. Conclusions

The results presented here demonstrated, for the first time, the inhibitory effect of Organoselenium Compounds on the development of dual-species biofilms of *Candida albicans* and *Candida krusei*, suggesting the potential of these compounds as promising antifungal medicine.

## Data Availability

Data is contained within the article.
